# Hybrid treat-and-repair strategy for large patent ductus arteriosus: a proof-of-concept case report

**DOI:** 10.1093/ehjcr/ytae354

**Published:** 2024-07-23

**Authors:** Naoki Tsuboya, Yoshihide Mitani, Hiroyuki Ohashi, Hirofumi Sawada, Masahiro Hirayama

**Affiliations:** Department of Pediatrics, Mie University Graduate School of Medicine, 2-174 Edobashi, Tsu City, Mie Prefecture 514-8507, Japan; Department of Pediatrics, Mie University Graduate School of Medicine, 2-174 Edobashi, Tsu City, Mie Prefecture 514-8507, Japan; Department of Pediatrics, Mie University Graduate School of Medicine, 2-174 Edobashi, Tsu City, Mie Prefecture 514-8507, Japan; Department of Pediatrics, Mie University Graduate School of Medicine, 2-174 Edobashi, Tsu City, Mie Prefecture 514-8507, Japan; Department of Pediatrics, Mie University Graduate School of Medicine, 2-174 Edobashi, Tsu City, Mie Prefecture 514-8507, Japan

**Keywords:** Patent ductus arteriosus, Pulmonary arterial hypertension, Treat and repair, Congenital heart disease, Case report

## Abstract

**Background:**

In cases of atrial septal defect with pulmonary arterial hypertension (PAH), a treat-and-repair strategy that adopts pulmonary vasodilator therapy and subsequent defect closure is postulated to be effective. However, this strategy has not been applied to the large patent ductus arteriosus (PDA) with PAH.

**Case summary:**

A 10-year-old girl with trisomy 21 was referred to our hospital for the treatment of a large PDA with PAH. Cardiac catheterization and angiography revealed a type C tubular PDA with a minimal diameter of 8.1 mm, an increase in mean pulmonary artery pressure (mPAP) of 60 mmHg, a ratio of pulmonary to systemic blood flow (Qp/Qs) of 2.7, and pulmonary artery resistance (Rp) of 7.1 U/m^2^. Because she was categorized in the grey zone for operability, we adopted a hybrid treat-and-repair strategy in which palliative surgical duct banding was performed before pulmonary vasodilator therapy to prevent excessive pulmonary blood flow and was followed by transcatheter closure of the PDA. Postoperatively, we confirmed the flow-restricted duct with a minimal diameter of 3.3 mm, decreased Qp/Qs 1.38, high mPAP 40 mmHg, and Rp 7.3 U/m^2^. Six months after treatment with macitentan and tadalafil, we confirmed a decrease in Rp 4.1 U/m^2^ as well as low Qp/Qs 1.12, which was low enough for the duct occlusion. The transcatheter occlusion of the surgically created type A conical duct was easily and safely performed. In the mid-term follow-up, favourable haemodynamics and improved exercise were confirmed.

**Discussion:**

This is the first proof-of-concept case report to show the successful hybrid treat-and-repair strategy for large PDA, which warrants further investigation.

**Learning points**

- The treat-and-repair approach for large unrestrictive patent ductus arteriosus (PDA) with severe pulmonary arterial hypertension (PAH) is challenging because of the risk of heart failure caused by high pulmonary blood flow under pulmonary vasodilator treatment.
- The present hybrid treat-and-repair approach, combined with palliative duct banding, has overcome such a risk as well as conferred the safer transcatheter closure of the residual shunt.
- The preset ‘proof-of-concept’ approach for large unrestrictive PDA with PAH warrants further studies and would be verified in the long-term follow-up.

## Introduction

Although a ‘treat-and-repair’ strategy in which pulmonary vasodilator is administered initially and subsequent defect closure is postulated to be effective in the cases of atrial septal defect with pulmonary arterial hypertension (PAH),^1,2^ this strategy has not been applied to large patent ductus arteriosus (PDA) with PAH because of excessive pulmonary blood flow due to pulmonary vasodilator therapy. Herein, we report a proof-of-concept case of catheter closure for large PDA with PAH in a patient who underwent palliative surgical duct banding initially followed by a treat and repair approach.

## Summary figure

**Table:** 

Time	Events
In infancy	Diagnosis of PDA
10 years of age	Diagnosis of PDA with PAH. Mean pulmonary artery pressure (mPAP) of 60 mmHg, the ration of pulmonary to systemic blood flow (Qp/Qs) of 2.69, and pulmonary vascular resistance (Rp) of 7.1 Wood units/m^2^.
5 months later	Palliative surgical duct banding. Mean PAP of 40 mmHg, Qp/Qs of 1.38, and Rp of 7.3 Wood units/m^2^.
7 months later	Started treatment with macitentan and tadalafil.
13 months later	Mean PAP of 36 mmHg, Qp/Qs of 1.12, and Rp of 4.5 Wood units/m^2^. Transcatheter occlusion of the duct.
20 months later	Mean PAP of 29 mmHg and Rp of 4.4 Wood units/m^2^.

## Case presentation

**Figure 1:**
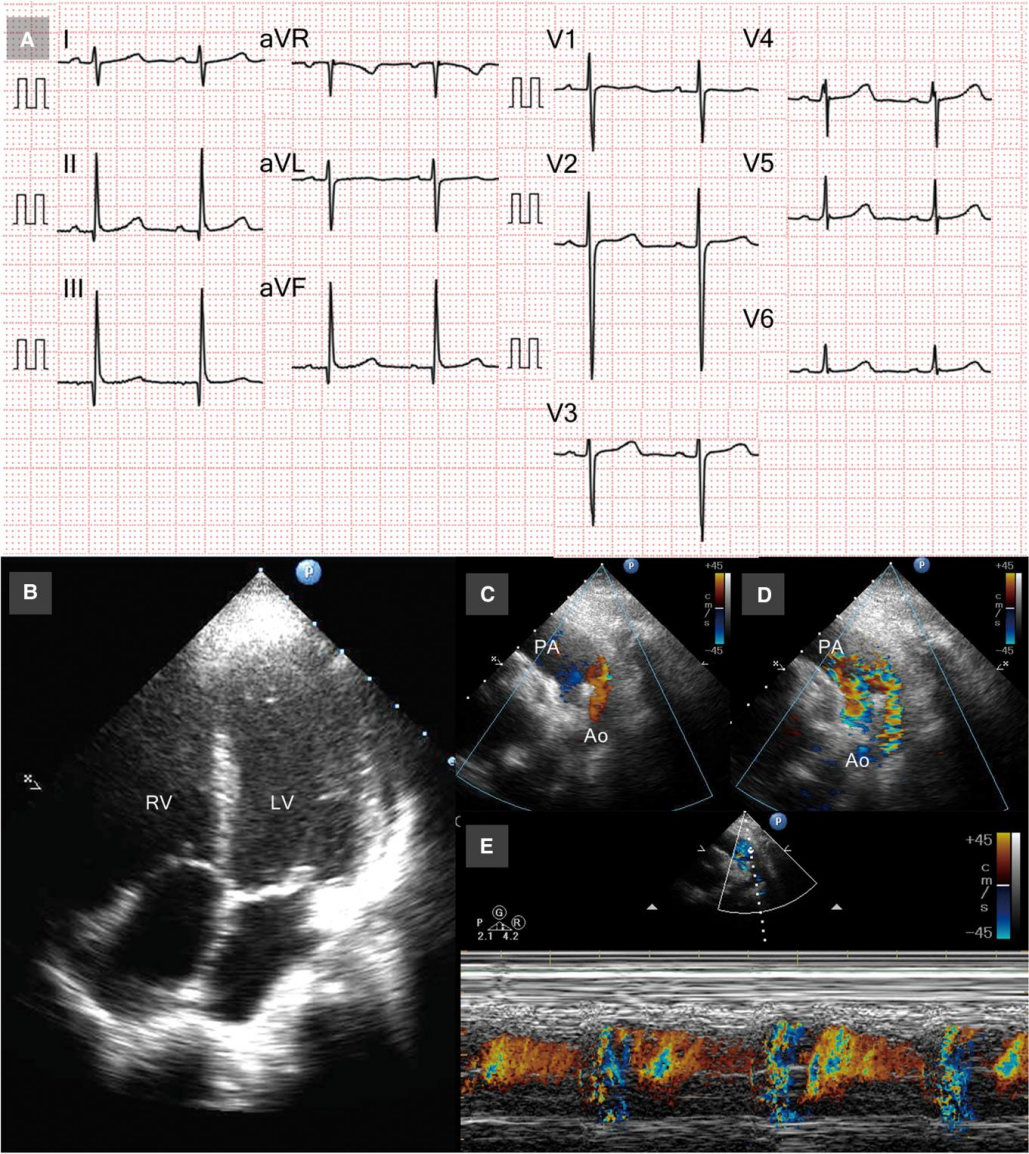
Electrocardiogram and echocardiogram images. Electrocardiogram when admission revealed no left or right ventricular hypertrophy (*A*). Echocardiogram showed well-balanced right ventricle and left ventricle with small pericardial effusion (*B*) and patent ductus arteriosus with bidirectional shunt (*C–E*). RV, right ventricle; LV, left ventricle; PA, pulmonary artery; Ao, descending aorta.

**Figure 2:**
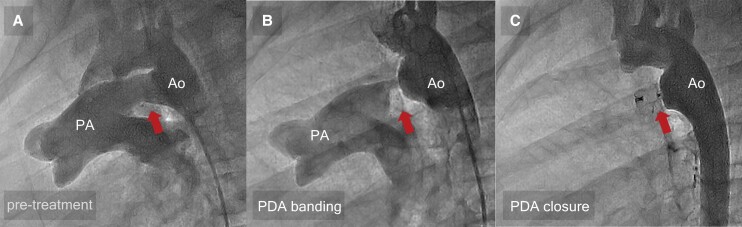
Aortography images. Aortography images of pre-treatment (*A*), post-patent ductus arteriosus banding (*B*), and post-patent ductus arteriosus closure (*C*). The pre-treatment imaging of the aortography showed a ‘tubular’-shaped patent ductus arteriosus with systemic-to-pulmonary shunt (*A*), but after patent ductus arteriosus banding, the form of the patent ductus arteriosus appeared ‘conical’ (*B*). After transcatheter closure using Amplatzer™ Duct Occluder I, no residual shunt was observed (*C*). The arrows indicate the patent ductus arteriosus area. Ao, aorta; PA, pulmonary artery; PDA, patent ductus arteriosus.

A 10-year-old girl with trisomy 21 was referred to our hospital for the treatment for large PDA with severe pulmonary hypertension. Although she was diagnosed with a large PDA with severe pulmonary hypertension in infancy, she was followed up conservatively with diuretics and angiotensin-converting enzyme inhibitors because of her family’s refusal of the surgery. Physical examination revealed no cyanosis (percutaneous oxygen saturation of 98% in the upper and lower extremities), continuous murmur (Levine 2/6 at the upper left sternal border), or no tachypnoea. Chest radiogram revealed an enlarged pulmonary artery with moderately increased pulmonary vascularity and cardiothoracic ratio of 58%. Electrocardiogram revealed no left or right ventricular hypertrophy (*Figure 1A*). Echocardiogram showed well-balanced right ventricle and left ventricle with small pericardial effusion (*Figure 1B*), moderate tricuspid valve regurgitation of 3.9 m/s, and large PDA with bidirectional shunt (*Figure 1C–E*). Angiographic image revealed a 13.6 mm long tubular arterial duct^3^ (type C in Krichenko classification), measuring ϕ9.2 mm on the pulmonary artery side, ϕ8.1 mm in the middle, and ϕ12.7 mm on the aortic side, which was regarded as having a high risk of device embolization during the transcatheter occlusion in the setting of pulmonary hypertension^4^ (*Figure 2A*). Cardiac catheterization revealed an increase in mPAP, the ratio of pulmonary to systemic blood flow (Qp/Qs), pulmonary vascular resistance (Rp), and Rp/systemic vascular resistance (Rs) (*Table 1*). The occlusion test and the acute pulmonary vasodilator test (AVT) with inhaled 20 ppm nitric oxide plus 100% O_2_ showed a decrease in mPAP and an increase in descending aortic pressure (dAoP) and the cardiac index (CI), with a minimal change in the Rp or Rp/Rs. She was diagnosed with large PDA associated with PAH, which is categorized in the grey zone for operability.^1,2,5,6^ We therefore adopted the ‘treat-and-repair’ strategy, in which palliative surgical arterial duct banding was performed before pulmonary vasodilator therapy for several months and was followed by transcatheter closure of the PDA. In the banding of the arterial duct, the duct was exposed through a left thoracotomy and banded at 10 mm from the aorta with a circumference of 15 mm (outer diameter ϕ4.8 mm) using a 0.6 mm thick and 5 mm wide polytetrafluoroethylene tape (W. L. Gore & Associates, Flagstaff, AZ, USA). The final diameter and the site of the duct banding were determined by considering the diameter of the device used for catheter closure. In the cardiac catheterization and angiography 10 days after the duct banding procedure, we confirmed the flow-restricted arterial duct, with the minimal duct diameter of ϕ3.3 mm, decreased pulmonary blood flow, and mPAP (*Figure 2B* and *Table 1*). Subsequently, we initiated macitentan and tadalafil. Cardiac catheterization 6 months after initiating the pulmonary vasodilators revealed a decrease in Rp and Rp/Rs in response to the acute vasodilator testing, which was low enough for the transcatheter occlusion.^1,2,6^ Such banding converted the shape of the duct to a ‘conical’ duct form, called type A (the smallest diameter ϕ2.8 mm, the aortic side ϕ17.4 mm) (*Figure 2B*). The transcatheter occlusion of the residual PDA was easily performed using the Amplatzer™ Duct Occluder I (ADOI) (Abbott Laboratories, Abbott Park, IL, USA) with a size of 8/6 mm (*Figure 2C*). The macitentan and tadalafil were continued. No apparent device-related adverse events were observed. Cardiac catheterization 8 months after PDA occlusion revealed a favourable haemodynamics (*Table 1*). Exercise tolerance improved after the duct occlusion.

**Table 1:** Trends in indices associated with pulmonary circulation evaluated by cardiac catheterization

	Pre-treatment			Post-PDA banding	6-month TDT and just before TCO		8 months after TCO	
	Rest	Occlusion	Occlusion + AVT		Rest	AVT	Rest	AVT
mPAP (mmHg)	60	39	32	40	36	32	29	21
Qp/Qs	2.69	1.0	1.0	1.38	1.12	1.22	1.0	1.0
CI (mL/min/m^2^)	2.25	2.99	4.03	2.97	4.61	7.90	4.02	4.60
Rp (U/m^2^)	7.1	9.0	5.2	7.3	4.5	1.9	4.4	2.6
Rp/Rs	0.30	0.37	0.33	0.43	0.40	0.29	0.33	0.22
dAoP (mmHg)	63	83	72	57	59	60	56	60

mPAP, mean pulmonary artery pressure; Qp/Qs, the ratio of pulmonary to systemic blood flow; CI, cardiac index; Rp, pulmonary vascular resistance; Rs, systemic vascular resistance; dAo, descending aortic pressure; TDT, targeting drug therapy; TCO, transcatheter occlusion; AVT, acute pulmonary vasodilator test.

## Discussion

Without proper management, large PDA with increased pulmonary flow leads to advanced pulmonary vasculopathy with high pulmonary artery resistance, in which the extreme form of the disease is Eisenmenger syndrome.^2^ Although the treat-and-repair strategy has been applied to atrial septal defect with increased Rp,^1,2^ it was unclear whether this strategy can be applied to large arterial duct with PAH. Since the administration of pulmonary vasodilators to patients with unrestrictive PDA may carry the risk of acute heart failure due to the excessive pulmonary blood flow, we overcame this issue by performing palliative duct banding in the present case. Furthermore, the palliative surgical duct banding gave us the opportunity to easily and safely occlude the remaining duct by percutaneous catheter occlusion using ADOI for the surgically created type A arterial duct. To the best of our knowledge, this is the first case report to show the successful treat and repair for large arterial duct after palliative duct banding, which was followed by transcatheter occlusion of the duct. The present ‘proof-of-concept’ case warrants further studies to establish a treat-and-repair approach for large PDA with the high pulmonary vascular resistance.

## Data Availability

The data underlying this article will be shared upon reasonable request to the corresponding author.

## Lead author biography

Naoki Tsuboya, MD, belongs to the Mie University Graduate School of Medicine Faculty of Paediatrics, works as a paediatric cardiologist at Mie University Hospital, and is engaged in basic research on pulmonary hypertension.
